# Enhancement of cutaneous immunity during aging by blocking p38 mitogen-activated protein (MAP) kinase–induced inflammation

**DOI:** 10.1016/j.jaci.2017.10.032

**Published:** 2018-09

**Authors:** Milica Vukmanovic-Stejic, Emma S. Chambers, Mayte Suárez-Fariñas, Daisy Sandhu, Judilyn Fuentes-Duculan, Neil Patel, Elaine Agius, Katie E. Lacy, Carolin T. Turner, Anis Larbi, Veronique Birault, Mahdad Noursadeghi, Neil A. Mabbott, Malcolm H.A. Rustin, James G. Krueger, Arne N. Akbar

**Affiliations:** a Division of Infection and Immunity, University College London, London, United Kingdom; b Laboratory for Investigative Dermatology, Rockefeller University, New York, NY; c Department of Dermatology, Royal Free Hospital, London, United Kingdom; d NIHR Biomedical Research Centre at Guy's and St Thomas's Hospitals and King's College London, Cutaneous Medicine and Immunotherapy, St John's Institute of Dermatology, Division of Genetics and Molecular Medicine, King's College London School of Medicine, Guy's Hospital, King's College London, London, United Kingdom; e Biomedical Sciences Institutes: Agency for Science, Technology and Research (A*STAR), Singapore; f Francis Crick Institute, London, United Kingdom; g Roslin Institute and Royal (Dick) School of Veterinary Studies, University of Edinburgh, Edinburgh, United Kingdom

**Keywords:** Aging, p38 mitogen-activated protein kinase, varicella zoster virus, inflammation, CBA, Cytometric Bead Array, CRP, C-reactive protein, DC, Dendritic cell, DEG, Differentially expressed gene, MAP, Mitogen-activated protein, T_RM_, Resident memory T, VZV, Varicella zoster virus

## Abstract

**Background:**

Immunity decreases with age, which leads to reactivation of varicella zoster virus (VZV). In human subjects age-associated immune changes are usually measured in blood leukocytes; however, this might not reflect alterations in tissue-specific immunity.

**Objectives:**

We used a VZV antigen challenge system in the skin to investigate changes in tissue-specific mechanisms involved in the decreased response to this virus during aging.

**Methods:**

We assessed cutaneous immunity based on the extent of erythema and induration after intradermal VZV antigen injection. We also performed immune histology and transcriptomic analyses on skin biopsy specimens taken from the challenge site in young (<40 years) and old (>65 years) subjects.

**Results:**

Old human subjects exhibited decreased erythema and induration, CD4^+^ and CD8^+^ T-cell infiltration, and attenuated global gene activation at the site of cutaneous VZV antigen challenge compared with young subjects. This was associated with increased sterile inflammation in the skin in the same subjects related to p38 mitogen-activated protein kinase–related proinflammatory cytokine production (*P* < .0007). We inhibited systemic inflammation in old subjects by means of pretreatment with an oral small-molecule p38 mitogen-activated protein kinase inhibitor (Losmapimod; GlaxoSmithKline, Brentford, United Kingdom), which reduced both serum C-reactive protein levels and peripheral blood monocyte secretion of IL-6 and TNF-α. In contrast, cutaneous responses to VZV antigen challenge were increased significantly in the same subjects (*P* < .0003).

**Conclusion:**

Excessive inflammation in the skin early after antigen challenge retards antigen-specific immunity. However, this can be reversed by inhibition of inflammatory cytokine production that can be used to promote vaccine efficacy and the treatment of infections and malignancy during aging.

## Graphical abstract

**Figure:**
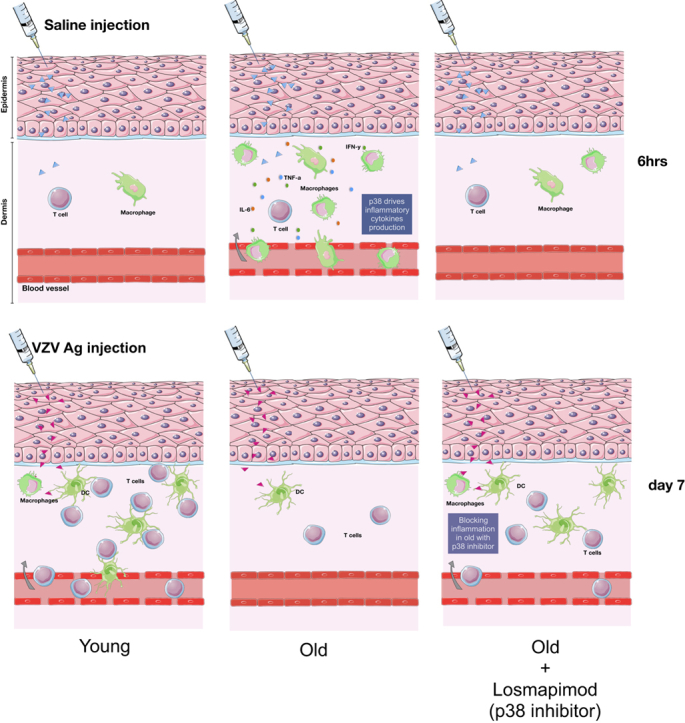

Older subjects have reduced immune function that predisposes them to an increased incidence of infection and malignancy.^1^, ^2^ In addition, vaccine efficacy against many pathogens is also reduced in these subjects.^3^ We developed a human experimental system to investigate antigen-specific immunity *in vivo* in which healthy volunteers are challenged intradermally to induce antigen-specific delayed-type hypersensitivity responses. This enabled the investigation of the kinetics and specificity of memory T-cell expansion and the interactions between different leukocytes after a single episode of immune stimulation *in situ*.^4^, ^5^, ^6^

Varicella zoster virus (VZV) is an α-herpesvirus that causes chickenpox. After resolution of the initial infection, VZV becomes latent within dorsal root ganglia but reactivates in older subjects causing herpes zoster (shingles).^7^, ^8^ During both primary infection and latent virus reactivation, the absence of T-cell immunity results in VZV-induced pathology.^9^, ^10^ Therefore decreased skin responsiveness to VZV challenge is a good model for investigating immune decline during aging.

Old subjects exhibited reduced erythema and induration (clinical response) after injection of a VZV skin test antigen that was correlated with decreased T-cell infiltration and proliferation in the skin. This was not due to defective macrophage activation^4^ or reduced inherent function of skin-resident memory T (T_RM_) cells.^11^ On the contrary, we identified a propensity of the skin of old but not young subjects to mount an overexuberant proinflammatory response on sterile challenge with a physiological saline solution. This was significantly inversely correlated with decreased VZV antigen responsiveness in the same subjects.

Previous studies demonstrated that systemic inflammation, as indicated by increased levels of serum IL-6, TNF-α, and C-reactive protein (CRP), are strong predictors for frailty and mortality during aging.^12^, ^13^ Ingenuity Pathway Analysis indicated a significant association between the inflammatory gene in the skin of old subjects with p38 mitogen-activated protein (MAP) kinase pathway activation (*P* = 1 × 10^−18^). We tested the hypothesis that the magnitude of the sterile proinflammatory response in the skin and reduced antigen-specific immunity in the same subjects was linked. To do this, we treated old human subjects with the oral p38 MAP kinase inhibitor Losmapimod (GlaxoSmithKline, Brentford, United Kingdom) for 4 days to inhibit proinflammatory cytokine production. This resulted in a significant reduction in CRP levels and peripheral blood monocyte secretion of IL-6 and TNF-α after stimulation *in vitro* but a significantly increased response to cutaneous VZV antigen challenge in the same subjects. Thus decreased VZV antigen challenge responsiveness in the skin of old subjects is related to excessive proinflammatory responses. Therefore anti-inflammatory intervention might be a strategy for boosting cutaneous immunity during aging.

## Methods

### Study design

This work was approved by the Ethics Committee of Queen Square (London, United Kingdom) and by the institutional review board (UCL R&D). Healthy young subjects (<40 years of age; n = 97; median age, 29 years) and old subjects (>65 years of age; n = 78; median age, 75.5 years) were recruited (see Tables E1 and E2 in this article's Online Repository at www.jacionline.org). Exclusion criteria are described in the Methods section in this article's Online Repository at www.jacionline.org. All volunteers provided written informed consent, and study procedures were performed in accordance with the principles of the Declaration of Helsinki.

### Skin tests

VZV antigen (BIKEN, the Research Foundation for Microbial Diseases of Osaka University, Osaka, Japan) was injected intradermally into sun-unexposed skin of the medial proximal volar forearm, according to the manufacturer's instructions. Induration, palpability, and the change in erythema from baseline were measured and scored on day 3, as described previously.^14^ A clinical score (range, 0-10) based on the summation of these parameters was then calculated.^14^ The injection site was sampled by means of skin biopsy at different times after injection with VZV skin test antigen.

### Losmapimod treatment

A subgroup of 18 old volunteers (8 male and 10 female subjects; age range, 65-77 years; median age, 69 years) were subjected to VZV antigen skin testing, as described above. Approximately 2 to 3 months later, volunteers received 15 mg of Losmapimod (GW856553) twice daily for 4 days (provided by GlaxoSmithKline under a Medical Research Council Industrial Collaboration Agreement). The dose of 15 mg of Losmapimod twice daily used in this study was chosen on the basis of the pharmacokinetic, pharmacodynamic, and safety profiles of Losmapimod observed in GlaxoSmithKline phase I and II studies.^15^

On day 4 of Losmapimod treatment, VZV skin test antigen was injected intradermally, and clinical scores were recorded 48 hours later, as before. A history of liver disease or increased liver transaminase levels (>1.5 times the upper limit of normal) and abnormal electrocardiographic results were additional exclusion criteria for this part of the study. Serum CRP levels were measured by using a high-sensitivity assay.^16^

To assess compliance, *ex vivo* whole-blood LPS stimulation assays were performed before and 4 days after Losmapimod treatment.^17^ Briefly, peripheral blood was cultured with LPS (0-1 mg/mL) for 24 hours (37°C in a 5% CO_2_ atmosphere). Levels of TNF-α and IL-6 in plasma were assessed by using the Cytometric Bead Array (CBA; BD, San Jose, Calif).

### Skin biopsies

Punch biopsy specimens (5 mm in diameter) from the site of antigen injection were obtained from young and old volunteers at various time points (as indicated) after VZV skin test antigen injection. Control skin punch biopsy specimens from normal (uninjected) forearm skin were also obtained. Biopsy specimens were frozen in OCT compound (Bright Instrument Company, Luton, Belgium), as previously described.^4^, ^11^ Six-micrometer sections were cut and left to dry overnight and then fixed in ethanol and acetone and stored at −80°C.

### Immunohistochemistry

Skin sections from normal, VZV skin test antigen–injected, or saline-injected skin were stained with optimal dilutions of primary antibodies, as previously described (see Table E3 in this article's Online Repository at www.jacionline.org).^4^, ^11^ The number of positively stained cells per square millimeter was counted manually by using computer-assisted image analysis (National Institutes of Health Image software 6.1; http://rsb.info.nih.gov/nih-image). Cell numbers were expressed as the mean absolute cell number counted within the frame.

### Immunofluorescence

Sections were stained with optimal dilutions of primary antibodies and followed by an appropriate secondary antibody conjugated to various fluorochromes, as previously described (see Table E4 in this article's Online Repository at www.jacionline.org).^4^, ^11^ The number of cells in 5 of the largest perivascular infiltrates present in the upper and middle dermis were selected for analysis, and an average was calculated.^18^ Macrophage images were imaged on the AxioScan Z1 slide scanner and Imaged on Zen Blue (Zeiss, Cambridge, United Kingdom).

### Skin biopsy digestion for flow cytometric analysis

Skin biopsy specimens (5 mm) were taken from normal and saline-injected skin (6 hours after injection) and disaggregated by means of overnight incubation (37°C in a 5% CO_2_ atmosphere) in 0.8 mg/mL collagenase IV (Sigma-Aldrich, St Louis, Mo) with 20% FCS. Single-cell suspensions were obtained by filtering the suspension through 100-, 70-, and 40-μm filters. Cells derived from skin biopsy specimens were assessed by using flow cytometric analysis on a BD Fortessa with FACSDiva software (BD Biosciences) and subsequently analyzed with FlowJo software (version X; TreeStar, Ashland, Ore). For details of antibodies used, see Table E5 in this article's Online Repository at www.jacionline.org.

### CBA

IL-6, IL-8, and TNF-α plasma concentrations were measured by using CBA (BD), according to the manufacturer's protocol. The lower limit of detection for each analyte was 1.5 pg/mL.

### Transcriptional analyses

Three-millimeter punch biopsy specimens were collected from the injection site 6 or 72 hours after injection with VZV antigen or normal saline and frozen immediately in RNAlater. Normal (uninjected) skin from the same site was collected as a control. Frozen tissue was homogenized, and total RNA was extracted from bulk tissue homogenates by using the RNeasy Mini Kit (Qiagen, Hilden, Germany). Details of gene expression analyses are provided in the Methods section in this article's Online Repository.

Where indicated, human skin-punch microarray data were combined with a large collection of other primary cell gene-expression data sets (745 individual microarray data sets) available from the Gene Expression Omnibus database on the same Affymetrix Human Genome U133 Plus 2.0 expression array platform (Affymetrix, Santa Clara, Calif). The entire collection of primary cell expression data are available through Gene Expression Omnibus accession number GSE49910. Full details of each primary cell data set have been published.^19^ Upstream regulator analysis was performed with Ingenuity Pathway Analysis (Qiagen).

### Statistics

Statistical analysis was performed with GraphPad Prism software (version 6.00; GraphPad Software, San Diego, Calif). Paired or unpaired *t* tests were used when data were normally distributed, and nonparametric tests were used when data were not normally distributed. The Kruskal-Wallis test was used to compare 3 or more unpaired groups, and a 2-tailed Mann-Whitney test was used when comparing only 2 unpaired groups. The Wilcoxon matched pairs test was used when comparing 2 groups of matched data. Two-way ANOVA was used to compare the effects of Losmapimod and LPS.

## Results

### Decreased response to VZV challenge in the skin during aging

**Fig 1:**
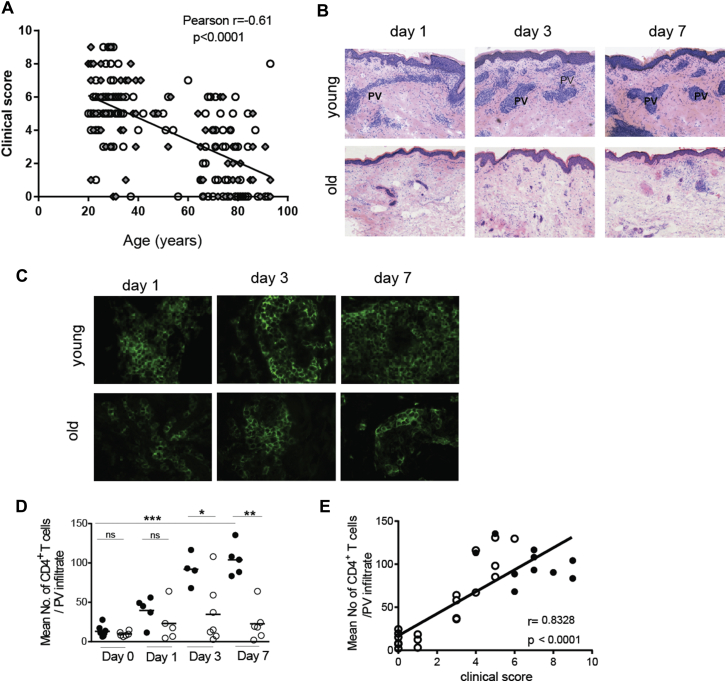
Cutaneous response to VZV antigen is reduced in old subjects. Healthy young and old volunteers were injected with VZV skin antigen. *Circles*, Female subjects; *diamonds*, male subjects. Clinical score at day 3 in response to VZV was calculated based on induration, palpability, and redness. **A,** Clinical score versus participant's age. **B,** Hematoxylin and eosin staining (×10 magnification). *PV*, Perivascular infiltrates. Five-millimeter punch biopsies were performed on days 0, 1, 3, or 7 after injection (with 4-7 volunteers per time point). **C,** Representative skin sections stained for CD4 (green; original magnification ×400). **D,** Collated data of T-cell numbers at different times after VZV injection in young and old volunteers. Each *symbol* represents the average number of CD4^+^ T cells within perivascular infiltrates for each subject (n = 4-7 per time point; Mann-Whitney test; *horizontal bar* represents the mean). **E,** Clinical score at 48 hours (peak clinical response) correlated with the number of CD4^+^ T cells in the perivascular infiltrate at the peak of cellular response on day 7 (n = 10 young *[solid circles]* and 22 old *[open circles]* subjects). *P < .05, **P < .01, and ***P < .001. *ns*, Not significant.

**Fig E1:**
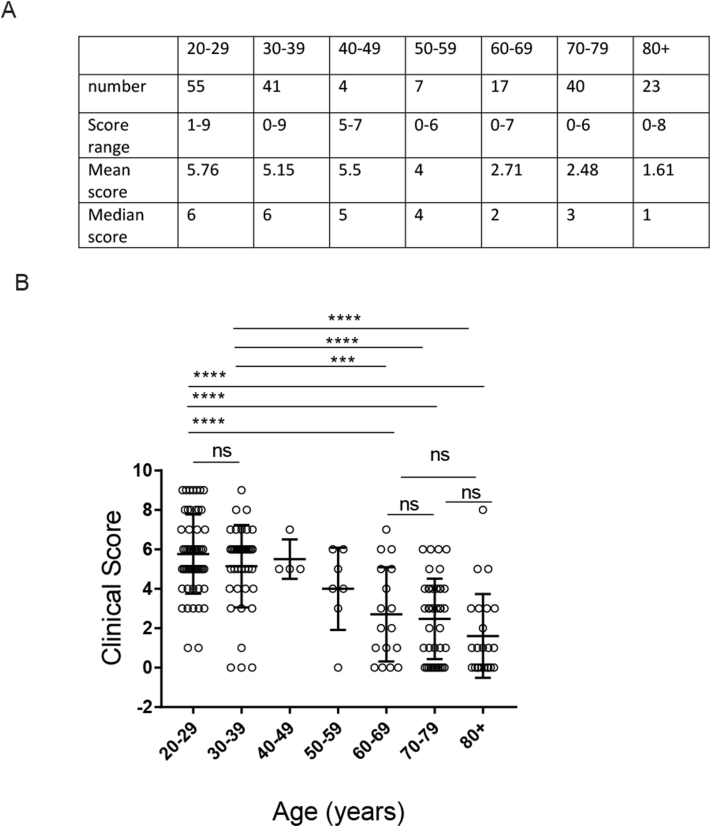


We investigated the cutaneous response of young (<40 years) and old (>65 years) volunteers to VZV antigen skin challenge. All volunteers had a prior history of chickenpox. At 72 hours after VZV injection, young subjects had obvious erythema and induration (clinical responses), whereas the clinical response of old subjects was significantly less and correlated inversely with increasing age (*P* < .0001, n = 184; young, n = 97; old, n = 78; middle age [40-65 years], n = 14; Fig 1, *A*; see Fig E1 in this article's Online Repository at www.jacionline.org; for participant details, see Tables E1 and E2). There were no differences in the response of male and female donors in the young and old age groups (*P* = .5, see Table E2). The decrease in clinical score was associated with decreased CD4^+^ T-cell accumulation at the site of VZV challenge in the skin at all time points investigated (Fig 1, *B-D*). There was a highly significant correlation between the clinical score (measured at 72 hours) and the extent of CD4^+^ T-cell accumulation in old subjects (measured at 7 days; Fig 1, *E*). We stratified old subjects into those who had a low skin response to VZV (clinical score, <4; 88% of old volunteers) and those with responses similar to those of young subjects with a clinical score of greater than 4 (86% of young volunteers).

**Fig E2:**
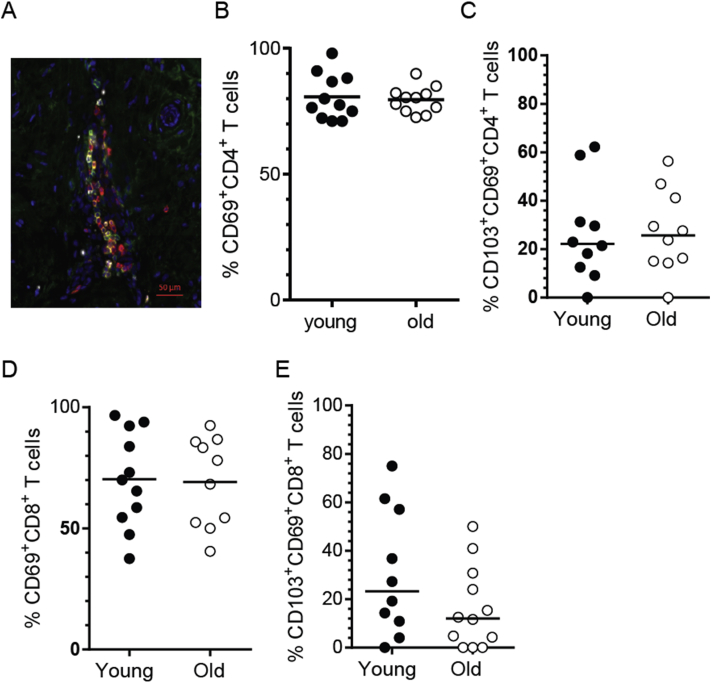


The decreased cutaneous response to VZV in old donors was not related to differences in numbers of resident memory CD4^+^ or CD8^+^ T cells defined by expression of CD69 alone or the combination of CD69 and CD103^20^, ^21^ in the skin of young and old subjects (see Fig E2 in this article's Online Repository at www.jacionline.org).^11^

### Reduced dendritic cell/T-cell interaction and T-cell proliferation after VZV challenge in old subjects

**Fig 2:**
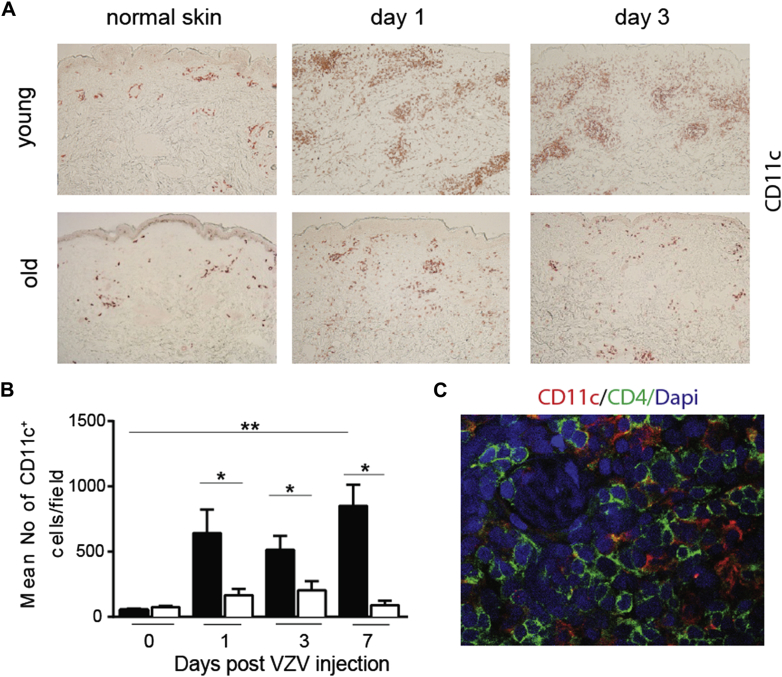
Perivascular cluster formation is reduced in the skin of old subjects. Five-millimeter punch biopsies were performed on days 0, 1, 3, or 7 after VZV injection (with 3-6 volunteers per time point). **A,** Representative staining of skin sections immunostained for CD11c (original magnification ×10). **B,** Cumulative data showing mean CD11c^+^ cell numbers per field (*solid bars*, young subjects; *open bars*, old subjects). Data are shown as means ± SEMs. *P < .05 and **P < .01. **C,** Representative staining showing CD11c^+^ DCs (red) and CD4^+^ T cells (green) in a perivascular cluster (representative young donor on day 3 after VZV injection, ×400 magnification). *Dapi*, 4′-6-Diamidino-2-phenylindole dihydrochloride.

Immune clusters containing both antigen-presenting cells and T cells (referred to as skin-associated lymphoid tissue) are generated in the skin during cutaneous immune responses.^22^ A highly significant increase in the number of CD11c^+^ dendritic cells (DCs) was observed in the skin of young but not old subjects at different times after VZV antigen challenge (Fig 2, *A* and *B*). DCs accumulate around blood vessels and form large perivascular clusters with CD4^+^ T cells (Fig 2, *C*) and, to a lesser extent, CD8^+^ T cells (data not shown). By 3 days after VZV antigen challenge, the majority of DCs within these infiltrates in the skin of young subjects were CD1c^−^ and expressed DC-lysosome–associated membrane glycoprotein, a marker of mature inflammatory DCs. In the skin of old subjects after VZV antigen challenge, DC infiltration was significantly reduced compared with that in the young group (Fig 2, *A* and *B*).

**Fig E3:**
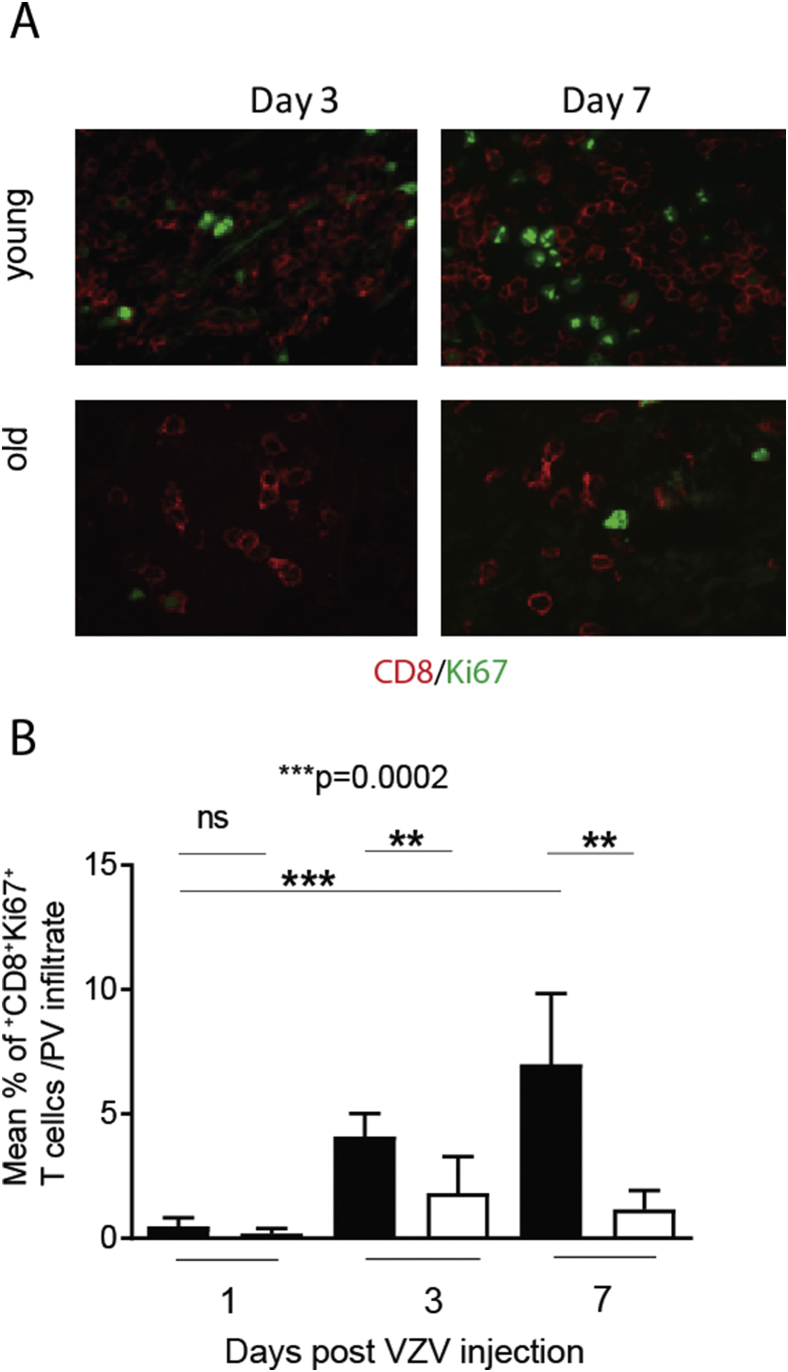


In young subjects proliferating (Ki67^+^) CD4^+^ T cells were undetectable in normal skin, but a significant increase was observed from 3 days after VZV antigen challenge compared with baseline values (see Fig E3, *A* and *B*, in this article's Online Repository at www.jacionline.org). Proliferation of CD8^+^ T cells was also observed, although to a lesser extent than for CD4^+^ T cells (see Fig E3, *C* and *D*). In contrast, in the skin of old subjects CD4^+^ and CD8^+^ T-cell proliferation was extremely low, even after 7 days of VZV antigen challenge (see Fig E3). This indicates that in young subjects the increased accumulation of T cells in the skin after VZV antigen challenge occurs in part from their proliferation at the site of injection.

**Fig E4:**
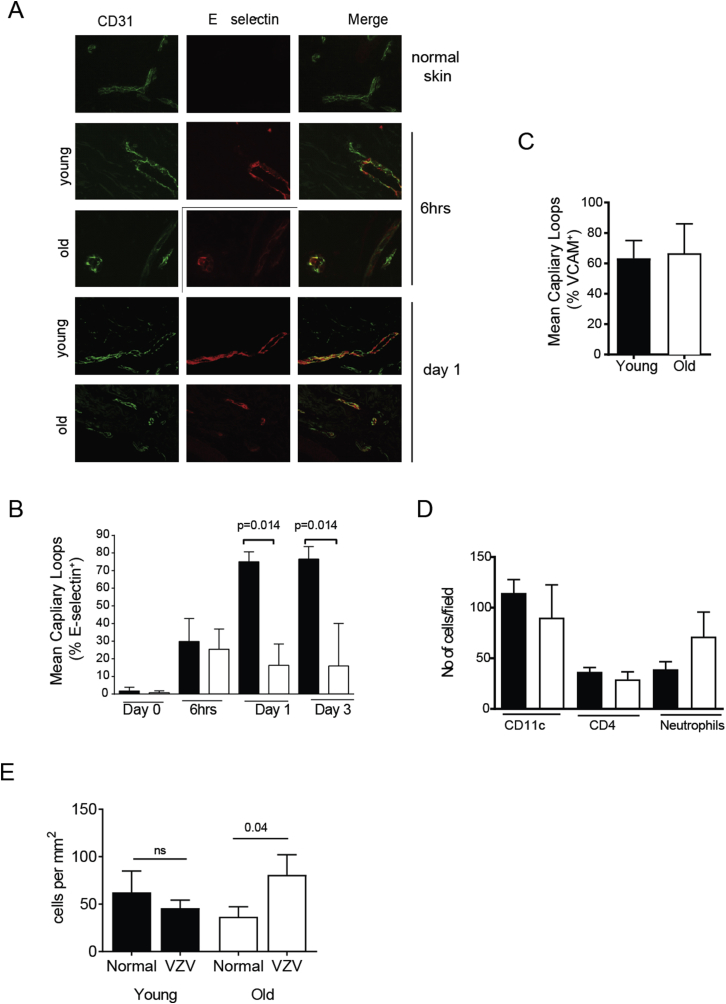


We investigated whether decreased endothelial cell activation contributed to the reduced T-cell infiltration in the skin of old subjects. At 6 hours after VZV antigen injection, 20% of the CD31^+^ capillary loops in young and old skin expressed both E-selectin and vascular cell adhesion molecule 1, levels of which were significantly greater than those observed in unchallenged (day 0) skin from either age group (see Fig E4 in this article's Online Repository at www.jacionline.org). This suggests that endothelial cells in old subjects are not defective and can be activated to the same extent as young subjects early in the response. At later time points, capillary loops in young subjects showed significantly increased expression of E-selectin when compared with those in old subjects (see Fig E4). Therefore reduced response to VZV antigen challenge was not due to defects in initiation of the response but was related to either active inhibition, lack of immune amplification at later stages, or both.

### Global decrease in the magnitude of gene expression in skin after VZV challenge in old subjects

**Fig 3:**
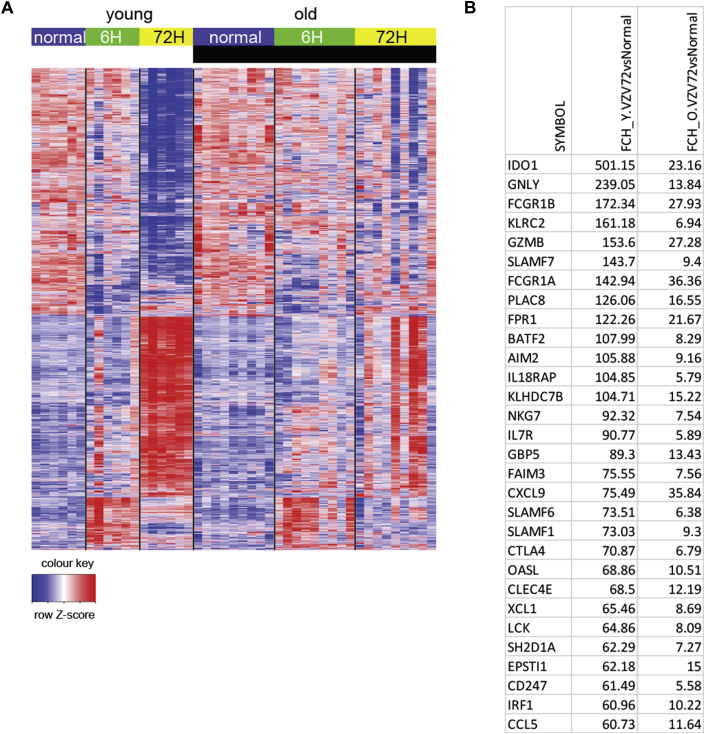
Transcriptomic analysis of skin from young and old subjects after VZV antigen challenge. Three-millimeter punch biopsy specimens were collected from old (n = 10) and young (n = 6) subjects at 6 and 72 hours after VZV injection. Normal skin punch biopsy specimens were collected from an additional group of young (n = 9) and old (n = 6) subjects. Total skin RNA was isolated, amplified, and hybridized to Affymetrix Human Genome U133 2.0 plus arrays. **A,** Heat map showing relative expression of DEGs between VZV-injected and normal skin from young *(left)* and old *(right)* subjects at a fold change *(FCH)* of greater than 2 and a false discovery rate of greater than 0.05 in normal/unmanipulated skin and skin 6 and 72 hours after VZV challenge in each group. For each gene, only the probe set with the largest average expression is shown. Unsupervised clustering was carried out by using Pearson correlation distance with the McQuitty agglomeration scheme of DEGs at 6 hours after VZV. **B,** The table shows the top 30 upregulated genes at 72 hours in young and old subjects compared with normal skin in each group.

We next performed global gene expression analyses to identify genes that might be associated with the decreased response to VZV antigen challenge in old subjects. In each young or old donor, skin punch biopsy specimens were taken from the site of either VZV antigen challenge (6 and 72 hours after injection) or saline injection (6 and 72 hours after injection; control) in the contralateral arm of the same subjects (resulting in a total of 4 biopsy specimens per subjects). Gene expression was compared with the signature in biopsy specimens taken from normal uninjected skin from 6 young and 9 old donors (Fig 3). In a previous study we showed that there was no evidence for inflammatory responses in either group at steady state.^11^

**Fig 4:**
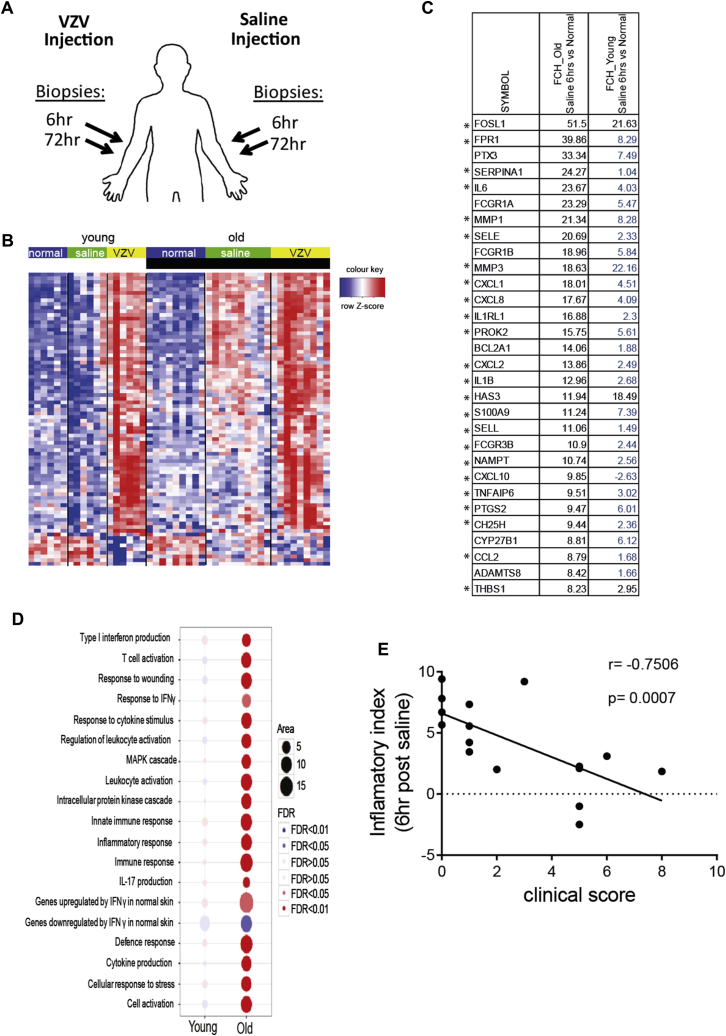
Comparison of global gene expression between normal, saline-injected, and VZV antigen–injected skin. **A,** Schematic representation of biopsy collection for transcriptional analysis. **B,** Heat map showing relative expression of DEGs (fold change *[FCH]* > 2 and false discovery rate > 0.05) between normal skin and saline-injected skin at 6 hours after treatment in young *(left)* and old *(right)* subjects. **C,** The table shows the top 20 upregulated genes at 6 hours in saline-injected skin from old and young subjects compared with normal skin. Genes not reaching statistical significance are indicated in blue. *Asterisks* indicate genes related to p38 MAP kinase signaling. **D,** Bubble plot shows expression of pathways in saline-injected skin versus normal skin. Kyoto Encyclopedia of Genes and Genomes (KEGG) and GO collection, as well as curated skin-related collection, were interrogated, and the most relevant pathways among them with an false discovery rate of less than 0.05 are presented. **E,** Inflammatory index was calculated for each subject (see the Methods section) and plotted against VZV clinical scores at 72 hours (young subjects, n = 6; old subjects, n = 10).

**Fig E5:**
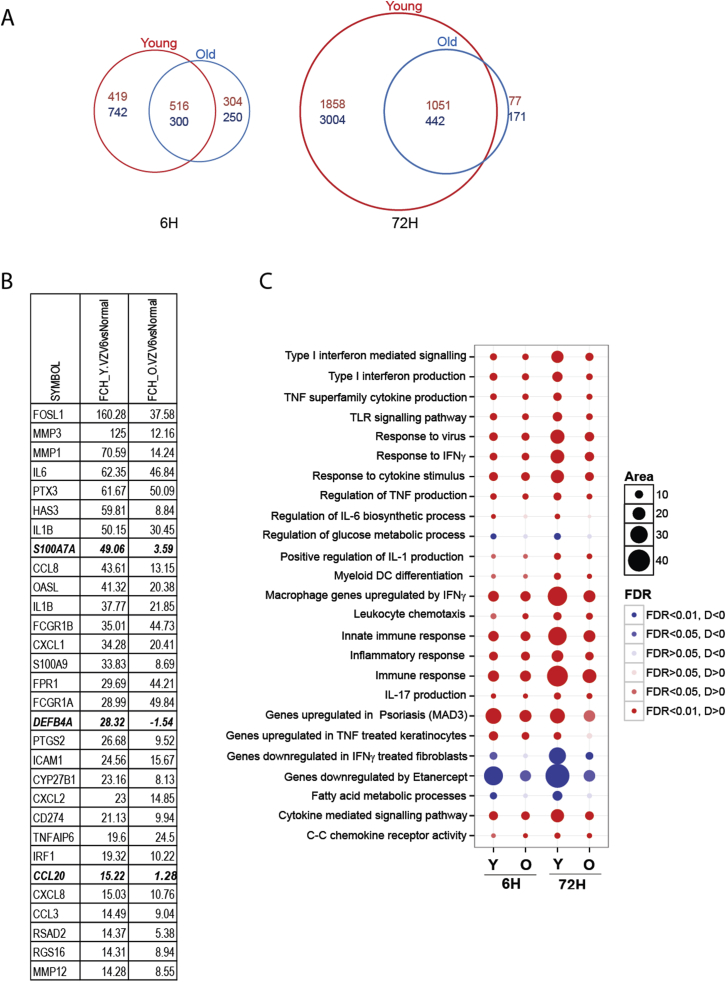


Six hours after VZV antigen challenge, there were 935 significantly upregulated and 1042 downregulated genes in the skin of young subjects and 820 upregulated and 550 downregulated genes in the old group (see Fig E5, *A*, in this article's Online Repository at www.jacionline.org). Although similar pathways were activated in the skin of young and old subjects at 6 hours after VZV antigen challenge, the magnitude of their expression was reduced in the old group (see Fig E5, *B* and *C*). At 72 hours after VZV antigen challenge, young subjects exhibited a strong transcriptional response that was considerably reduced in the old group (>5000 differentially expressed genes [DEGs] in the young and 666 DEGs in the old group, Fig 3). The top 30 genes that are significantly differentially expressed are shown in Fig 4, *B*, indicating that the same genes are upregulated in both groups but that the expression was reduced in the old subjects, indicating attenuated immune amplification. Genes associated with T-cell and DC activation, including *ITGAX* (which encodes CD11c), *CD2*, *CD28*, *CD69*, *CD83*, *CD86*, *EOMES*, *ICOS*, and *STAT1*, were more highly expressed in the skin at 72 hours of VZV challenge in the young group compared with the old group (see Table E6 in this article's Online Repository at www.jacionline.org). There was activation of signaling pathways associated with immune responses, inflammation and immune response to viruses, and pathways induced by type I interferons and IFN-γ signaling in young but not old subjects (see Fig E5).

### Saline injection induces an inflammatory response in old donors, which correlates inversely with cutaneous VZV response

Sterile saline solution was a physiological control injected into the contralateral arms of the same subjects who received VZV skin antigen challenge (Fig 4, *A*). In young subjects saline injection had a negligible effect on gene expression compared with normal skin (30 DEGs at 6 hours after injection; Fig 4, *A* and *B*). However, in old subjects saline injection induced significant early expression of numerous inflammatory genes (856 DEGs; false discovery rate < 0.05; fold change > 2; Fig 4, *A* and *B*; see Table E7 in this article's Online Repository at www.jacionline.org), including *IL6*, *CXCL8*, and *PTX3*, and also genes indicative of myeloid cell activation, including *CXCL2*, *IL1B*, *ICAM1*, and *FCGR3A* (Fig 4, *B*, and see Table E7).

**Fig E6:**
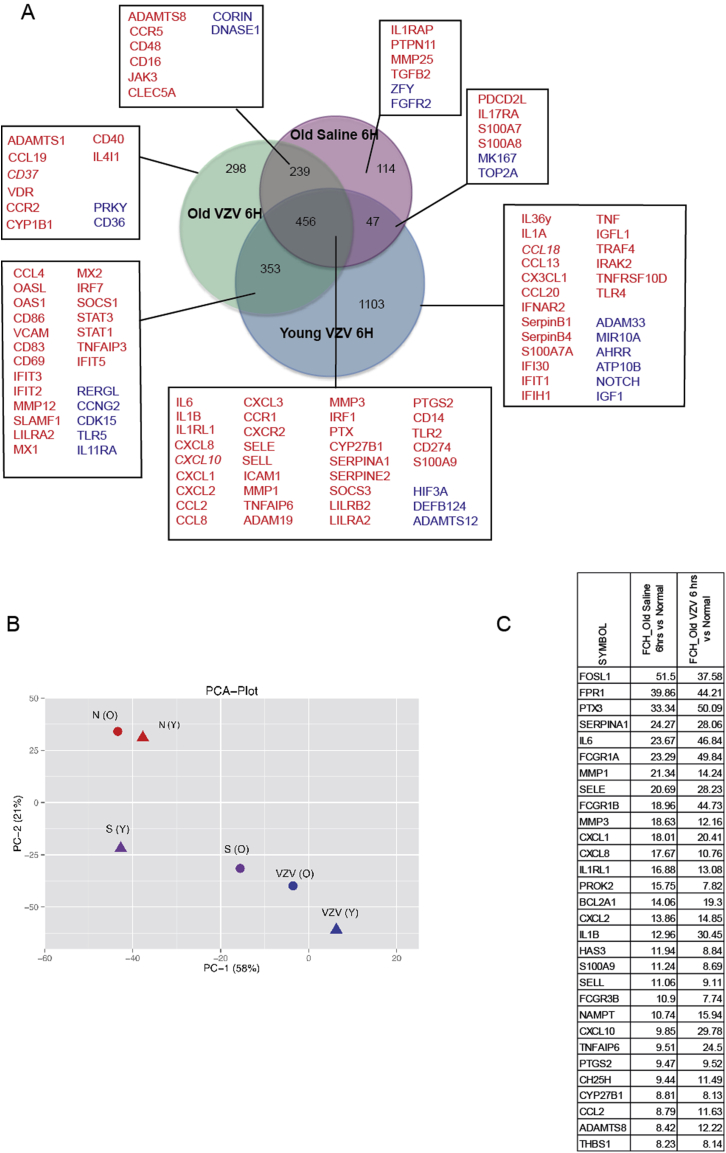


Using Ingenuity Pathway Analysis, we found a significant association with predicted p38 MAP kinase pathway activation (*P* = 1 × 10^−18^) when the genes that were upregulated after saline injection (6 hours) in old skin were compared with unchallenged control old skin. The majority of the top 30 genes activated after 6 hours of saline injection (Fig 4, *C*, asterisks) are induced by p38 MAP kinase signaling or are regulators of p38 MAP kinase activation. Furthermore, pathway analysis suggested that the response to saline in the skin of old subjects involved the activation of inflammatory pathways relating to type I interferon production, TNF-α signaling, MAP kinase activation, IFN-γ responses, and IL-17 production (Fig 4, *D*). Principal component analysis demonstrated that gene expression in response to saline injection in young and old subjects was distinct (see Fig E6, *B*) and that a large number of the genes that were upregulated by 6 hours after saline injection in old skin were also induced by VZV antigen challenge at the same time point (Fig 4, *C*, and see Fig E6). These data indicate that the early transcriptional response to VZV antigen challenge in the skin of old subjects includes an inflammatory component that might not be specific for the antigen itself.

**Fig E7:**
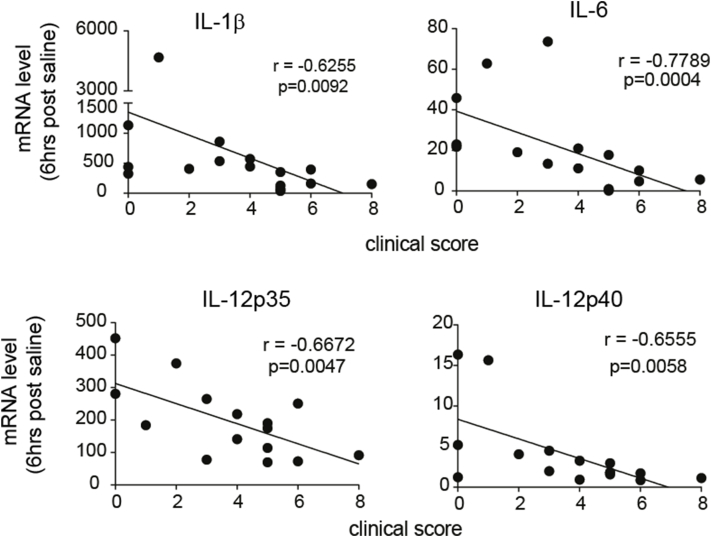


We investigated whether the propensity to exhibit sterile inflammatory responses at 6 hours after nonspecific (saline-induced) inflammation in aging skin was directly associated with decreased clinical response to VZV challenge in the contralateral arm of the same subjects at 48 hours. To address this, the expression levels of 384 genes designated as positive regulators of the inflammatory response (see Table E8 in this article's Online Repository at www.jacionline.org) were compared in the skin of young (n = 6) and old (n = 10) subjects at 6 hours after saline injection. By using gene set variation analysis, each subject was assigned a numeric score (denoted as the inflammatory index) based on the variation in expression of all these inflammatory genes. A highly significant inverse correlation was observed when the inflammatory index value to saline injection for each subject was plotted against the clinical response to intradermal VZV antigen challenge in the contralateral arm (Fig 4, *E*). A similar and significant inverse correlation was observed between expression levels of IL-1β, IL-6, IL-12p35, and IL-12p40, as determined by using quantitative RT-PCR and a subject's clinical response to VZV antigen challenge were compared (see Fig E7 in this article's Online Repository at www.jacionline.org). This indicates an association between the propensity to exhibit early sterile inflammation and reduced responses to VZV antigen challenge in the skin of the same old subjects *in vivo*.

### Nonspecific inflammation induced by saline injection is associated with mononuclear phagocytes

**Fig 5:**
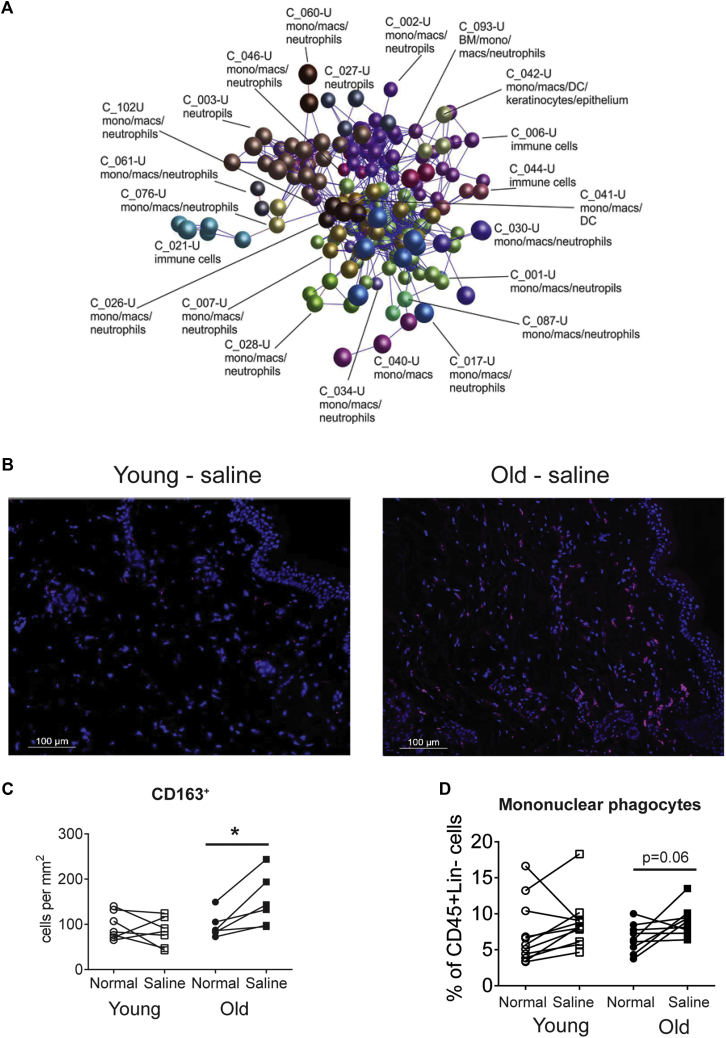
Identification of a monocyte/macrophage-related gene expression signature in saline-injected aged skin. **A,** Transcriptomic analysis using the tool BioLayout Express^3D^ of genes upregulated in the skin of elderly human subjects 6 hours after saline treatment, which clusters together in a large network of monocyte/macrophage-related genes (*C_*, cluster no.; nodes represent individual genes, and edges represent Pearson correlations > 0.7). **B** and **C,** Representative images of CD163-stained saline-injected skin from skin from young and old subjects (CD163 is pink, Dapi blue; Fig 5, *B*) and cumulative data of CD163^+^ cells in paired analysis from normal and saline-injected skin at 6 hours (Fig 5, *C*; n = 4-5 per age group). *P < .05. **D,** Frequency of mononuclear phagocytes determined as being CD45^+^ Lineage^−^ (CD3^−^, CD19^−^, CD20^−^, and CD56^−^) and HLA-DR^+^ and either CD14^+^ and/or CD16^+^ expressed as a percentage of CD45^+^ Lineage^−^ cells in young *(open symbols)* and old *(solid symbols)* subjects before and after saline, as assessed by using flow cytometry. Data are assessed by using the paired *t* test.

The DEGs identified at 6 hours after saline injection (false discovery rate < 0.05, fold change > 2; see Table E2) were imported into BioLayout Express^3D^ to identify the cell type that might contribute to the increased proinflammatory response to saline injection in aging skin (see the Methods section and Fig 5, *A*). Comparison of the mean cellular expression profiles of the gene clusters derived from this network graph suggested that many of the genes within them were strongly associated with cells of the monocyte/macrophage lineage (Fig 5, *A*, and see Table E9 in this article's Online Repository at www.jacionline.org).

**Fig E8:**
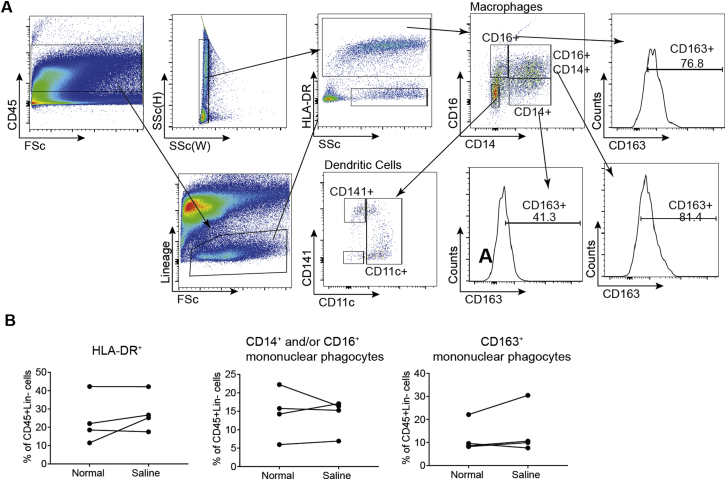


Furthermore, immunohistologic analysis also demonstrated a significant increase in the number of CD163^+^ mononuclear phagocytes in the skin of old subjects within 6 hours of saline injection (Fig 5, *B* and *C*). This was confirmed by using multiparameter flow cytometry, in which we identified significantly increased proportions of HLA-DR^+^CD14^+^ mononuclear phagocytes in old compared with young skin biopsy specimens 6 hours after saline injection (Fig 5, *D*, and see Fig E8 in this article's Online Repository at www.jacionline.org). This rapid increase in the frequency of CD14-expressing mononuclear phagocytes was probably a result of recruitment from the blood because these cells were not in cycle (data not shown).^23^ The increase in “inflammatory” monocytes in old subjects was transient and coincided with the transient sterile inflammatory response that was only observed at 6 but not 24 hours after saline injection. A similar significant transient increase in numbers of mononuclear phagocytes was observed when old subjects were injected with VZV (see Fig E4, *E*).

### Short-term p38 MAP kinase blockade improves clinical response to VZV skin challenge in older subjects

**Fig 6:**
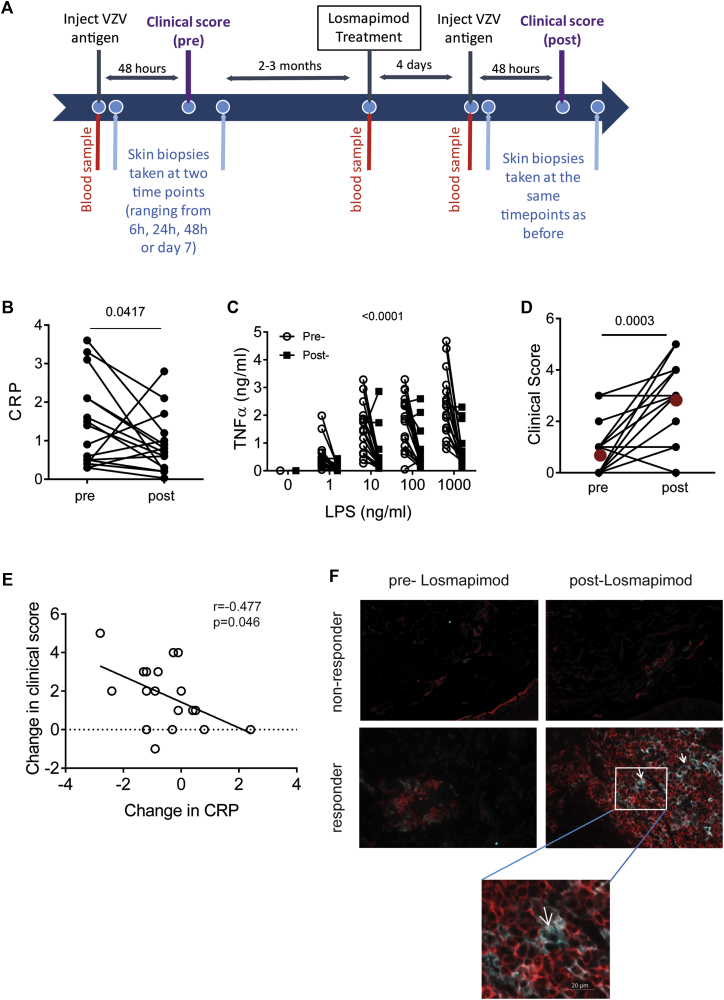
Effects of Losmapimod treatment on VZV response in the skin. **A,** Responses to VZV skin challenge were investigated in old subjects (n = 18, 8 male and 10 female subjects) before and after Losmapimod treatment (15 mg administered twice daily for 4 days). **B,** Serum CRP levels before and after Losmapimod treatment (n = 18; *P* = .04, Wilcoxon paired test). **C,** Whole-blood LPS stimulation was performed before and after Losmapimod treatment, and TNF-α production was measured by using CBA (LPS: *P* < .0001 and Losmapimod: *P* < .0001, 2-way ANOVA [n = 18]). **D,** Clinical score was measured at 48 hours after VZV antigen challenge before and after Losmapimod (*P* = .0006, Wilcoxon paired test; *red symbols* indicate the mean). **E,** Correlation between change in serum CRP level and change in clinical score after Losmapimod treatment (Pearson correlation). **F,** Representative images of skin sections collected 7 days after VZV injection stained for CD4 (red) and CD11c (pale blue) before and after Losmapimod treatment in one of the subjects with an increased clinical score in response to VZV improved after Losmapimod treatment (*top and bottom right panels*) and one of the subjects whose clinical score remained low after Losmapimod treatment (*top* and *bottom left panels*). *White arrows* indicate a DC interacting with surrounding T cells.

**Fig E9:**
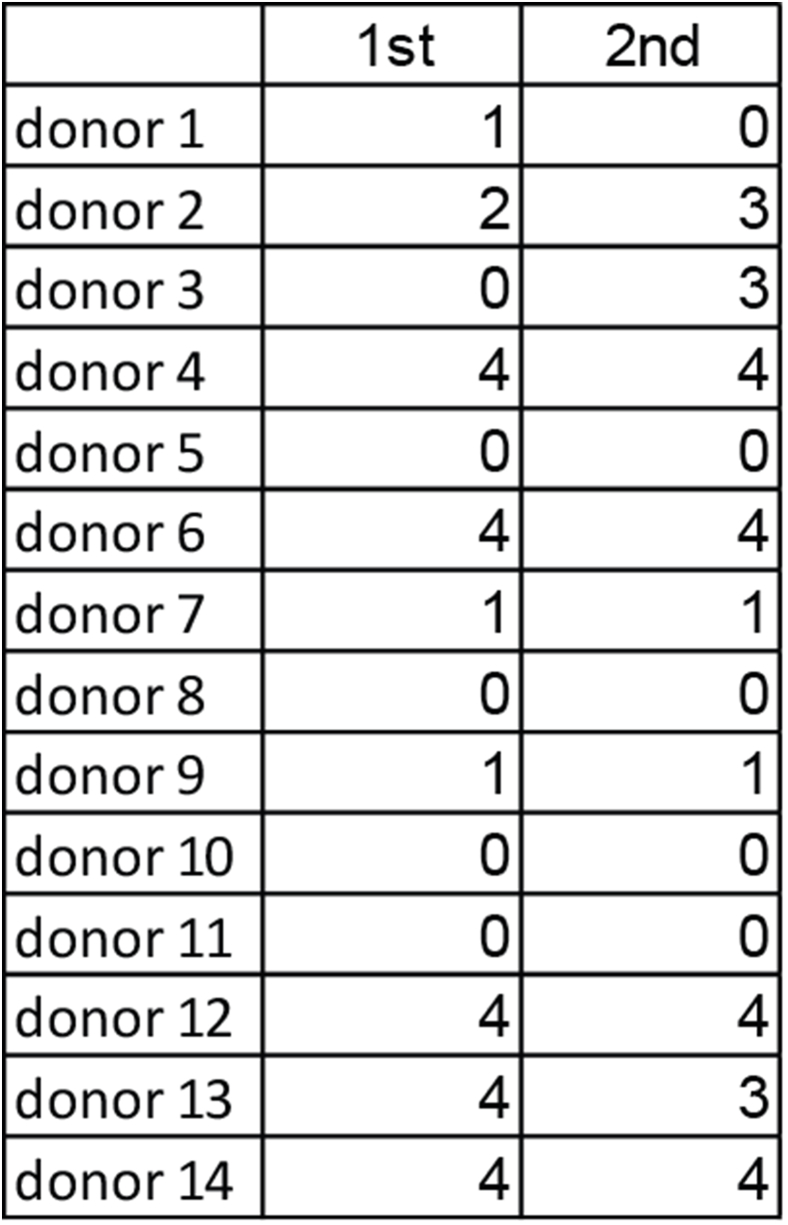


We tested the hypothesis that excessive proinflammatory cytokine secretion is driven by p38 MAP kinase signaling in mononuclear phagocytes early in the skin response. To do this, we treated 18 healthy old volunteers who had a low previous skin response to VZV challenge (clinical score, <4) with Losmapimod, a potent and selective oral p38 MAP kinase inhibitor, before VZV antigen rechallenge in the skin (Fig 6, *A*).^15^, ^16^ These subjects were investigated 2 to 3 months after the first skin test and pretreated with the drug for 4 days before rechallenge with VZV antigen in the skin. In parallel studies we showed that rechallenge of old volunteers with VZV skin test antigens did not significantly boost their original clinical scores (n = 14, *P* = .58, see Fig E9 in this article's Online Repository at www.jacionline.org).

**Fig E10:**
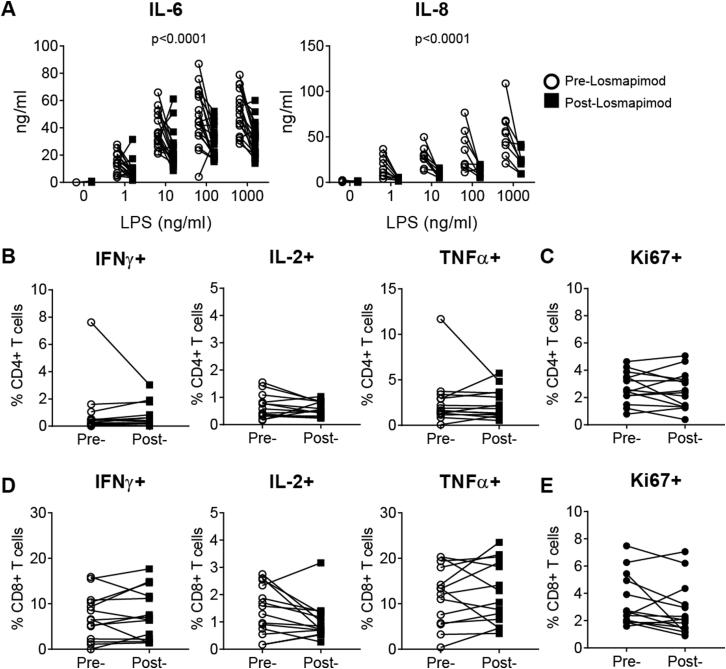


CRP production in the liver is upregulated by p38 MAP kinase–dependent cytokines, such as IL-1 and IL-6.^24^, ^25^ Serum CRP levels were significantly reduced after Losmapimod pretreatment (*P* = .04, n = 18; Fig 6, *B*). In addition, TNF-α, IL-6, and IL-8 production by LPS-stimulated PBMCs from the same donors was also significantly reduced after Losmapimod pretreatment (Fig 6, *C*, and see Fig E10 in this article's Online Repository at www.jacionline.org). In contrast, Losmapimod pretreatment significantly enhanced the clinical response to VZV antigen challenge in the skin of 13 of 18 old subjects (n = 18, *P* = .0006; Fig 6, *D*). This increase in clinical score to VZV challenge correlated with the decrease in CRP levels in serum in the same subjects (Fig 6, *E*). Losmapimod pretreatment had no effect on CD4^+^ or CD8^+^ T-cell function in response to CD3 and IL-2 stimulation in the same donors, as defined by cytokine expression (IFN-γ, IL-2, and TNF-α) or proliferation, as defined by Ki67 expression (see Fig E10). Histological assessment of biopsy specimens collected from 4 old subjects with an increased clinical response after Losmapimod treatment showed that there was a significant increase in the number of CD11c^+^ DCs in the perivascular infiltrates (*P* = .04) that were associated with increased numbers of CD4^+^ T cells in immune clusters (representative experiment shown in Fig 6, *F*, top and bottom right panels). These clusters were not found in 4 of the subjects who did not respond to Losmapimod treatment (Fig 6, *F*, top and bottom left panels). These clusters resembled those found after VZV challenge of skin in young subjects (Fig 2). This shows that p38 MAP kinase inhibition significantly reduced proinflammatory responses and that this was associated with enhancement of antigen-specific immune responses in the skin of old subjects *in vivo*.

## Discussion

The cutaneous recall response to intradermal antigen challenge is a manifestation of immune memory, and this reaction decreases with age.^4^, ^26^, ^27^, ^28^ We have investigated reasons for this decrease to explain the increased incidence of cutaneous infection and malignancy during aging.^2^, ^29^ Early inflammation (erythema and induration in 48 hours) is required to initiate the cascade of events leading to optimal cellular infiltration in the skin that occurs later (peak at 7 days).^14^ Therefore an unexpected observation was that excessive inflammation during the early phase after VZV challenge hinders the amplification of the response in old subjects. The response to VZV in older subjects is not inhibited from the outset because the endothelium of old and young subjects is activated equally at 6 hours, and gene expression at this time is similar. Furthermore, the decreased response after VZV antigen challenge in these subjects was not due to intrinsic changes in the functionality of cutaneous T_RM_ cells or macrophages because these cells from both age groups were equally responsive when isolated from skin and activated *in vitro*.^4^, ^11^

In other studies increased systemic inflammation has been shown to have a negative effect on the cutaneous recall response to *Candida* species antigens^30^; however, this study did not investigate the response of old subjects or events that occur in the skin itself. The reduced efficacy of vaccination has also been linked to excessive inflammation for influenza,^31^ yellow fever,^32^ tuberculosis,^33^ and hepatitis B^34^ vaccines. Furthermore, inflammatory macrophages in patients with chronic artery disease suppress T-cell activation and expansion *in vitro*, and this is associated with defective VZV-specific T-cell immunity in the peripheral blood of these patients.^35^ The proposed mechanism for this inhibition involves the upregulation of the inhibitory receptor ligand programmed death ligand 1 on the inflammatory macrophages that inhibit function of programmed cell death protein 1–expressing T cells.^35^ This suggests that the infiltration of inflammatory monocytes that express programmed death ligand 1 during sterile inflammation might block early activation of cutaneous T_RM_ cells because this latter population expresses significantly higher levels of PD-1 during aging.^11^

Type I interferon has been shown to interfere with antigen-specific T-cell responses, and excessive levels of these mediators impair the clearance of both viral and mycobacterial infections in mice *in vivo*.^36^, ^37^ In the present study we also found a strong type I interferon signature in the skin of old subjects after saline injection, although other inflammatory pathways were also upregulated. The effect of excessive inflammation on inhibition of antigen-specific T-cell function is particularly important for aging because older subjects have widespread low-grade systemic inflammation termed “inflammaging”^12^ that is linked to expression of inflammasome gene modules that might underpin clinical frailty and immune dysfunction.^13^

It is not clear why saline injection induces early but transient inflammation in the skin of old subjects (observed at 6 but not 24 hours after injection). However, NaCl itself is proinflammatory and has been shown to induce T_H_17 cells while conversely inhibiting forkhead box protein 3^+^ regulatory T-cell function^38^, ^39^ and to also activate inflammatory cascades in monocytes and bone marrow–derived macrophages *in vitro*.^40^, ^41^ The saline control that we used in the current study contained 0.9% NaCl, which is similar to the concentration used in the diluent of the VZV skin test antigen (0.68% NaCl). Therefore a component of the transcriptional response of old subjects to VZV antigen would also include a response to NaCl in the diluent that might hinder the induction of antigen-specific immunity in old subjects. This response is not observed in young subjects. We identified mononuclear phagocytes as the source of the saline-induced cutaneous inflammation.

Many of the inflammatory mediators induced by saline injection in older subjects were linked directly or indirectly to activation of the p38 MAP kinase pathway. Many pharmaceutical companies have tested small-molecule p38 MAP kinase inhibitors in human subjects *in vivo* in phase I, II, and III trials to block inflammatory diseases/disorders.^42^ Although most trials with p38 MAP kinase inhibitors were discontinued because of hepatotoxicity after long-term treatment (>3 months) and adaptation of cell-signaling pathways, leading to reduced drug efficacy,^43^ these inhibitors do not show evidence of toxicity in the short-term (weeks) in human subjects *in vivo.* Therefore we treated old subjects who were not responsive to cutaneous VZV antigen challenge with Losmapimod (GW856553), a selective, reversible, competitive inhibitor of p38 MAP kinase, to test the hypothesis that reducing inflammation in the skin would enhance antigen-specific cutaneous immunity. The key observation was that Losmapimod pretreatment significantly enhanced the cutaneous response to VZV in older subjects. Although our previous studies have shown that that p38 inhibition can enhance T-cell proliferation *in vitro*,^44^, ^45^, ^46^ in the current study Losmapimod treatment did not affect peripheral blood T-cell proliferation or cytokine production after stimulation with CD3/IL-2. Thus the enhancement of cutaneous immunity is likely to be due to the anti-inflammatory effects of the drug.

This raises the question of whether the short-term inhibition of p38 MAP kinase signaling and/or inhibition of inflammation would also enhance immunity in other tissues. An interesting possibility is that this would be a strategy to improve vaccine efficacy that is decreased in aging subjects.^3^, ^47^ Another point to consider is that increasing the strength of adjuvants to enhance vaccine responses during aging might be counterproductive if they further increase inflammatory responses, and it might be important to stratify old vaccinees on the basis of their baseline inflammatory responses in the future.^13^ Our study might appear to challenge the concept that antigen-specific immunization is more successful in the presence of an adjuvant that is designed to increase inflammatory responses. However, although adjuvants can enhance induction of immunity in draining lymph nodes, excessive inflammation that is present at the site of the effector phase of a response might inhibit T-cell responsiveness. This could be a mechanism to protect against pathology induced by excessive immune stimulation in the tissues. Furthermore, excessive inflammation is detrimental for cancer progression,^48^ and the temporary inhibition of inflammation in this situation might be a strategy for boosting immunotherapy in these patients. Although the current challenge is to identify the optimal way to reduce excessive inflammation and to enhance immunity in aging human subjects, it is serendipitous that some drugs that might do this have already been developed and therefore can be repurposed.

## Supplementary data

Online Repository text

Table E1

Table E2

Table E3

Table E4

Table E5

Table E6

Table E7

Table E8

Table E9
