# Supplementary material for: Enhancement of cutaneous immunity during aging by blocking p38 mitogen-activated protein (MAP) kinase–induced inflammation
Source: J Allergy Clin Immunol. 2018 Sep;142(3):844–56. doi: 10.1016/j.jaci.2017.10.032 (PMC6127037; doi:10.1016/j.jaci.2017.10.032)
Supplement: Online Repository text [file mmc1.docx]

**List of Supplementary material:**

**Tables:**

Supplementary Table 1. Information on age, gender and clinical score of participants recruited into the study

Supplementary table 2. Detailed information on gender and clinical score of participants recruited into the study.

Supplementary Table 3: Antibodies used in immunohistochemistry

Supplementary Table 4: Antibodies used in immunofluorescence

Supplementary Table 5: Antibodies used for Flow-cytometric analysis

Supplementary Table 6. VZV injected vs normal skin differentially expressed genes

Supplementary Table 7. Saline injected vs normal skin differentially expressed genes

Supplementary Table 8. List of 384 genes (positive regulators of inflammation) used in the inflammatory index

Supplementary table 9. Contents of clusters described in Fig 5A.

**Figures**:

Supplementary Figure 1. Clinical response to VZV antigen challenge in different age groups.

Supplementary Figure 2. Phenotype of CD4 and CD8 T cells resident in normal skin of young and old individuals.

Supplementary figure 3. Proliferation of CD8 T cells following VZV antigen challenge is reduced in the old.

Supplementary Figure 4. Activation of dermal endothelium at site of VZV challenge

Supplementary Figure 5. Pathway analysis of gene expression in young and old skin at 6 and 72 h post VZV antigen challenge.

Supplementary Figure 6. Overlap of differentially expressed genes (DEG) in the skin of young and old individuals in the skin after injection of saline or VZV antigen.

Supplementary Figure 7. Inflammatory response induced by saline injection inversely correlates with the response to VZV antigen challenge

Supplementary Figure 8. Saline injection increases the frequency of HLA-DR+ cells and mononuclear phagocytes in old but not young individuals.

Supplementary figure 9. Repeat skin testing with VZV skin antigen does not affect the clinical response in old individuals.

Supplementary Figure 10. Effect of Losmapimod treatment on immune function

**Supplementary text:**

**Methods:**

**Participant exclusion criteria:** Individuals with history of neoplasia, immunosuppressive disorders or inflammatory skin disorders were excluded from this investigation. Furthermore, we excluded individuals with co-morbidities that are associated with significant internal organ or immune dysfunction including heart failure, severe COPD, diabetes mellitus and rheumatoid arthritis and individuals on immunosuppressive regimes for the treatment of autoimmune or chronic inflammatory diseases (e.g. oral glucocorticoids, methotrexate, azathioprine and cyclosporin). We did not exclude volunteers with a history of uncomplicated hypertension or hypercholesterolaemia as this would have prevented the majority of ageing volunteers from participating in this study. The blood pressure and cholesterol level were not specifically measured for each volunteer, but those volunteers taking medication for a previously confirmed diagnosis of hypertension or hypercholesterolaemia were identified.

**PBMC stimulation:** PBMCs were isolated as standard and then subsequently stored at -80oC. The PBMCs were defrosted, counted and then cultured overnight at 5x105 cells/ml with plate bound anti-CD3 (1µg/ml) and IL-2 (50IU/ml) for eighteen hours at 37oC with 5% CO2. Brefeldin A (5µg/ml) was added two hours into the incubation. The cells were removed and cell surface stained with the following antibodies CD3, CD4, CD8 (clones UCHT1, SK3, SK9 respectively; BD) and Live/Dead after two washed the cells were fixed and permeabilised in Foxp3 staining buffers (as per the manufacturer’s instructions; eBiosciences) and intracellularly stained with the following antibodies: IFNγ, IL-2, TNFα and Ki67 (clones 4S.B3, MQ1-17H12, Mab11 and Ki-67 respectively; Biolegend. Samples were acquired on the BD Symphony (BD Biosciences) and were subsequently analysed using FLowJo Version X (Treestar).

**Transcriptional analysis of skin biopsies:** Target amplification and labelling was performed according to standard protocols using Nugen Ovation WB Kit. RNA was hybridized to Affymetrix Human Genome U133 2.0 plus arrays. Affymetrix gene chips were scanned for spatial artefacts using the Hirshlight package^1^. Gene expression measures were obtained using the GCRMA algorithm^2^ and was modelled using mixed-models in R’s limma framework. Differences between groups were estimated from this model and its significance assessed using the moderated (paired/unpaired) t-test. Resulting P-values were adjusted for multiple hypotheses using the Benjamini-Hochberg procedure. Gene set variation analysis (GSVA)^3^ was used to obtained the per-pathway scores for each patient and sample; using a collection of skin-specific pathways curated by the Krueger lab.

Network analysis of the genes expressed within skin biopsies was performed as described^4^. Briefly, normalized, nonlog-transformed, annotated, gene-expression data were imported into BioLayout Express^3D^ (www.biolayout.org), a tool designed specifically for the visualization of large gene-expression network graphs^5^. Network graphs were then created using a Pearson correlation coefficient cut-off threshold of *r* = 0.95. Each network graph was then clustered into groups of genes sharing similar profiles using the Markov clustering algorithm. The graphs were then explored to understand the biological significance of the gene clusters, identify those expressed by the young and old skin samples and their functional relationships to the other cell populations represented.

**Figure legends:**

**Supplementary Figure 1. Effect of age on clinical response to intradermal injection of VZV antigen.** Healthy young and old volunteers were injected with 0.02 ml VZV skin test antigen and clinical score based on a combination of extent of induration, palpability and redness at the injection site was measured at day 3 post challenge. Volunteers were split into age groups and mean, median and range f clinical scores calculated (A). Graph shows mean ± SEM for each age group. Mann-Whitney test was used to compare changes between age groups

.**Supplementary Figure 2. Phenotype of CD4 and CD8 T cells resident in normal skin of young and old individuals**. Sections of normal skin were immunostained to detect CD4, CD8, CD69, CD103 using an indirect immunofluorescence method. (A). Representative image of normal skin immunostained for CD4 (green), CD69 (red) and CD103 (white). (B) The proportion of CD4^+^ cells expressing CD69 in young and old skin (n=11). (C) The proportion of CD4^+^CD69^+^ cells expressing CD103 in young and old skin (n=10). (D) The proportion of CD8^+^ cells expressing CD69 in young and old skin (n=11). (E) The proportion of CD8^+^CD69^+^ cells expressing CD103 in young and old skin (n=10). For B-E the line indicates the mean.

**Supplementary Figure 3. Proliferation of T cells following VZV antigen challenge is reduced in the old.**  (A) Representative immunostaining of ki67 expression in skin afterday 3 and 7 post-VZV injection (Ki67 green; CD4 red). (B) Cumulative data showing the frequency of CD4^+^ cells expressing Ki67 per perivascular infiltrate (young - filled bars, old- open bars). (C) Representative immunostaining on days 3 and 7 post-injection: Ki67 (green) and CD8 (red). (D) Cumulative data of the percentage of CD8^+^ cells expressing Ki67 per perivascular infiltrate in each donor. Data shown as mean ± SEM. * = p<0.05, ** = p<0.01, *** = p<0.001.

**Supplementary Figure 4.** **Activation of dermal endothelium at site of VZV challenge** Immunofluorescence staining for CD31 and E-selectin or VCAm1 expression was performed on skin sections from biopsies taken from normal skin and 6 h, day 1 and day 3 after cutaneous challenge with VZV antigen from young and old volunteers (n=4-5 per age group at each time point). The number of double positive staining vessels expressed as a proportion of the total number of vessels in the superficial and mid-dermis of each section was used for analysis for each individual. (A) Representative images are shown CD31 (green) and E-selectin (red). (B) cumulative data (p values indicated, Mann Whitney test). (C) Expression of VCAM1 on CD31^+^ capillary loops 6hrs after VZV injection. (D) Healthy young and old volunteers (n=5) were injected with 0.02 ml VZV skin antigen test and 5 mm punch biopsies were performed 6hrs post injection. Skin sections were immunostained with CD11c, CD4 and neutrophil elastase and the number of positive cells was counted per field. For B-D data shown as mean ± SEM. (E) Normal and VZV (6 hours post-injection) paired skin biopsies were assessed for mononuclear phagocyte numbers by immunofluorescence staining utilising HLA-DR, CD14 and CD16. Any cell that was HLA-DR+ and CD14+ and/or CD16+ was defined as being a mononuclear phagocyte, analysis was performed in young (black, n=5) and old (white, n=5). For E data was assessed by a paired t-test * = p<0.05..

**Supplementary Figure 5.** **Pathway analysis of gene expression in young and old skin at 6 and 72 h post VZV antigen challenge** . Differentially expressed genes between VZV injected and normal skin in young or old at FCH>2 and FDR>0.05. Unsupervised clustering was carried out using Pearson correlation distance with Mcquitty agglomeration scheme. (A) Venn diagrams show numbers of DEG at 6 h following VZV antigen injection compared to normal skin. Up-regulated genes are shown in red, down-regulated genes in blue. (B) Table shows top 30 up-regulated genes at 6 h in young, genes not significantly up-regulated in old skin are indicated in bold italics. (C)**.** Bubble plot representing the overall representation of relative gene expression in VZV-injected skin versus normal skin. KEGG and GO collection, as well as a curated skin-related collection were interrogated and the most relevant pathways amongst them with FDR<0.05 are presented. The area of each circle is proportional to the differences in the GSVA-derived pathway scores between VZV-injected and normal skin in each group. Colours indicate the direction of dysregulation red (up) and blue (down). Colour intensity represents the strength of the dysregulation determined by FDR.

**Supplementary Figure 6.** **Overlap of differentially expressed of genes** (**DEG) in the skin of young and old individuals in the skin after injection of saline or VZV antigen.** A selection of differentially expressed genes of interest are indicated for each (red, up-regulated and blue, down-regulated). (B) Principal component analysis of global gene expression in normal skin and 6 h after injection with saline or VZV antigen. (C) Table shows top 30 up-regulated genes at 6 h in saline and VZV injected skin.

**Supplementary Figure 7. Inflammatory response induced by saline injection inversely correlates with the response to VZV antigen challenge.** The expression of individual inflammatory genes in the skin 6 h after saline was compared by qRT-PCR analysis and plotted against the clinical score following VZV antigen injection at 48 hours.

**Supplementary Figure 8. Frequency of HLA-DR^+^ cells and mononuclear phagocytes following saline injection** 5mm punch biopsies were collected from normal or injected skin and digested overnight to provide single cell suspension. (A) Gating strategy employed to identify mononuclear phagocytes and dendritic cells in human skin; CD45+ lineage cocktail negative single cells were identified, subsequently, HLA^-^DR^+^ CD14^+^ and/or CD16^+^ were mononuclear phagocytes and HLA-DR+CD14-CD16- were dendritic cells (DCs) either CD141^+^ or CD11c^+^ DCs. (B) cumulative data of mononuclear phagocyte populations 24 hours post-saline injection. (C) Phenotype of mononuclear phagocytes in the young and old donors (CD14^+^CD16^-^ grey, CD14^+^CD16^+^ white, CD16^+^CD14^-^ black). * = p<0.05

**Supplementary figure 9. Repeat skin testing with VZV skin antigen does not affect the clinical response in old individuals.** 14 individuals with clinical score below 4 were re-challenged with VZV in the skin 2-5 months after the original skin test. Clinical score for both skin tests are shown in the table (p>0.5).

**Supplementary figure 10. Effect of Losmapimod treatment on immune function (**A). Whole blood LPS stimulation was performed pre- and post-Losmapimod treatment in the same donors, and IL-6 and IL-8 was production measured by CBA (LPS p<0.0001, Losmapimod p<0.0001, Two way ANOVA n=18). PBMCs were stimulated overnight with CD3 and IL-2 were assessed by flow cytometric analysis in CD4+ T cells (B) for intracellular cytokine expression and (C) Ki67 expression and additionally in CD8 T cells (D) for intracellular cytokine expression and (E) Ki67 expression pre- and post-losmapimod treatment (white circles and black squares respectively). Figure B-E were assessed by a paired t-test and no significant difference was observed.

References:

1. Suarez-Farinas M, Haider A, Wittkowski KM. "Harshlighting" small blemishes on microarrays. BMC Bioinformatics 2005; 6:65.

2. Wu Z, Irizarry RA. Preprocessing of oligonucleotide array data. Nat Biotechnol 2004; 22:656-8; author reply 8.

3. Hanzelmann S, Castelo R, Guinney J. GSVA: gene set variation analysis for microarray and RNA-seq data. BMC Bioinformatics 2013; 14:7.

4. Mabbott NA, Baillie JK, Brown H, Freeman TC, Hume DA. An expression atlas of human primary cells: inference of gene function from coexpression networks. BMC Genomics 2013; 14:632.

5. Freeman TC, Goldovsky L, Brosch M, van Dongen S, Maziere P, Grocock RJ, et al. Construction, visualisation, and clustering of transcription networks from microarray expression data. PLoS Comput Biol 2007; 3:2032-42.
