# Supplementary material for: Enhancement of cutaneous immunity during aging by blocking p38 mitogen-activated protein (MAP) kinase–induced inflammation
Source: J Allergy Clin Immunol. 2018 Sep;142(3):844–56. doi: 10.1016/j.jaci.2017.10.032 (PMC6127037; doi:10.1016/j.jaci.2017.10.032)
Supplement: Table E1 [file mmc2.docx]

|  | Old female | Old male | Young female | Young male |
| --- | --- | --- | --- | --- |
| number | 47 | 31 | 56 | 41 |
| Age range | 65-93 | 65-93 | 20-39 | 20-39 |
| Average age | 75.7 | 77.5 | 28.3 | 29.6 |
| Median age | 74 | 77 | 29 | 29 |
| Score range | 0-8 | 0-6 | 0-9 | 0-9 |
| Mean score | 2.3 | 1.9 | 5.5 | 5.5 |
| Median score | 2 | 1 | 5 | 6 |
