# Supplementary material for: Enhancement of cutaneous immunity during aging by blocking p38 mitogen-activated protein (MAP) kinase–induced inflammation
Source: J Allergy Clin Immunol. 2018 Sep;142(3):844–56. doi: 10.1016/j.jaci.2017.10.032 (PMC6127037; doi:10.1016/j.jaci.2017.10.032)
Supplement: Table E2 [file mmc3.docx]

Supplementary Table 2:

|  | young | middle | old |
| --- | --- | --- | --- |
| number | 97 | 14 | 78 |
| Age range | 20-39 | 41-64 | 65-93 |
| Median age | 29 | 52 | 75.5 |
| Gender | 56F/41M | 8F/6M | 47F/31M |
| Score range | 0-9 | 0-7 | 0-8 |
| Mean score | 5.5 | 4.5 | 2.18 |
| Median score | 6 | 5 | 2 |
