# Supplementary material for: Enhancement of cutaneous immunity during aging by blocking p38 mitogen-activated protein (MAP) kinase–induced inflammation
Source: J Allergy Clin Immunol. 2018 Sep;142(3):844–56. doi: 10.1016/j.jaci.2017.10.032 (PMC6127037; doi:10.1016/j.jaci.2017.10.032)
Supplement: Table E3 [file mmc4.docx]

| **Antibody name** | **Clone** | **Company** |
| --- | --- | --- |
| CD11c | B-ly6 | BD Bioscience |
| CD4 | SK3 | BD Bioscience |
| CD163 | 5C6-FAT | Acris |
| DC-LAMP | 104.G4 | Beckman Coulter |
| Neutrophil elastase | NP75 | Dako |

Supplementary Table 3:
