# Supplementary material for: Enhancement of cutaneous immunity during aging by blocking p38 mitogen-activated protein (MAP) kinase–induced inflammation
Source: J Allergy Clin Immunol. 2018 Sep;142(3):844–56. doi: 10.1016/j.jaci.2017.10.032 (PMC6127037; doi:10.1016/j.jaci.2017.10.032)
Supplement: Table E4 [file mmc5.docx]

Supplementary Table 4:

| **Antibody name** | **Clone** | **Company** |
| --- | --- | --- |
| CD4 | SK3 or YNB46.1.8 | BD Bioscience |
| CD8 | RPA-T8 | BD Bioscience |
| Ki67 - FITC | B56 | BD Bioscience |
| CD31 - FITC | WM59 | BD Bioscience |
| CD11c | B-ly6 | BD Bioscience |
| Ki67 - FITC | B56 | BD Bioscience |
| CD69 | FN50 | Biolegend |
| CD103 | 2G5.1 | Thermofisher |
| Foxp3 - Biotin | PCH101 | eBioscience |
| PD-1 | NAT105 | Abcam |
| E-selectin | ENA1 | Abcam |
| CD163 | RM3/1 | Abcam |
