# Supplementary material for: Enhancement of cutaneous immunity during aging by blocking p38 mitogen-activated protein (MAP) kinase–induced inflammation
Source: J Allergy Clin Immunol. 2018 Sep;142(3):844–56. doi: 10.1016/j.jaci.2017.10.032 (PMC6127037; doi:10.1016/j.jaci.2017.10.032)
Supplement: Table E5 [file mmc6.docx]

| **Antibody name** | **Clone** | **Company** |
| --- | --- | --- |
| CD11c | 3.9 | Biolegend |
| CD14 | HCD14 | Biolegend |
| CD16 | 3G8 | Biolegend |
| CD19 | HIB19 | Biolegend |
| CD20 | 2H7 | Biolegend |
| CD56 | HCD56 | Biolegend |
| CD163 | GHI/61 | Biolegend |
| HLA-DR | 104.G4 | BD Biosciences |
| CD3 | UCHT1 | BD Biosciences |
| CD45 | 2D1 | BD Biosciences |
